# Body Condition of Reproductive and Non-Reproductive Broad-Snouted Caiman Females

**DOI:** 10.3390/ani14010001

**Published:** 2023-12-19

**Authors:** Evangelina V. Viotto, Pamela M. L. Leiva, Sofía E. Pierini, Melina S. Simoncini, Joaquín L. Navarro, Carlos I. Piña

**Affiliations:** 1 Centro de Investigación Científica y de Transferencia Tecnológica a la Producción, Consejo Nacional de Investigaciones Científicas y Técnicas, Provincia de Entre Ríos-Universidad Autónoma de Entre Ríos, España 149, Diamante CP 3105, Argentina; viotto.evangelina@uader.edu.ar (E.V.V.); pierini.sofia@uader.edu.ar (S.E.P.); melinasimoncini22@yahoo.com.ar (M.S.S.); 2 Proyecto Yacaré-Laboratorio de Zoología Aplicada: Anexo Vertebrados, Facultad de Humanidades y Ciencias, Universidad Nacional del Litoral, Aristóbulo del Valle 8700, Santa Fe CP 3000, Argentina; 3 Facultad de Ciencia y Tecnología, Universidad Autónoma de Entre Ríos, Tratado del Pilar 314, Diamante CP 3105, Argentina; 4 Centro de Zoología Aplicada, Facultad de Ciencias Exactas, Físicas y Naturales, Universidad Nacional de Córdoba, Rondeau 798, Córdoba CP 5000, Argentina; joaquin.navarro@unc.edu.ar; 5 Instituto de Diversidad y Ecología Animal-Consejo Nacional de Investigaciones Científicas y Técnicas, Universidad Nacional de Córdoba, Rondeau 798, Córdoba CP 5000, Argentina

**Keywords:** body condition index, *Caiman latirostris*, crocodile, reproduction, K-Fulton, scaled mass index (SMI), energetic investment

## Abstract

**Simple Summary:**

Using size and weight data from wild-caught adult broad-snouted caiman females, we calculated and compared two body condition indices (K-Fulton and SMI) with the objective of distinguishing reproductive from non-reproductive females. To achieve this, we looked for a threshold (size-dependent) that separated the body condition of females that were strictly reproductive from those that were not. Reproductive females showed better body condition than non-reproductive females. However, the minimum condition was not a determinant in separating reproductive from non-reproductive females, although it was possible to differentiate approximately 70% of the reproductive from the non-reproductive females. Therefore, we were able to categorize females >79 cm as females that will reproduce, despite not being in adequate condition, and <79 cm as females that will require a good body condition to reproduce. This information is of utmost importance when making decisions for the management or use, as well as for the conservation, of the species.

**Abstract:**

In this work, we calculated the body condition indices, K-Fulton and scaled mass index (SMI), of reproductive and non-reproductive *Caiman latirostris* adult females as an indication of stored energy. We considered 87 adult females captured from 2001 to 2018, both reproductive and non-reproductive. The body condition was calculated considering two scenarios: (a) only the weight of the female, and (b) the sum of the weight of the female and the average dry weight of her nest. We tested the difference in body condition between reproductive and non-reproductive females. We also evaluated the minimal body condition required to guarantee that females above it are reproductive by drawing a line that separated the body condition of strictly reproductive individuals from those that may or may not be reproductive. Reproductive females had better body condition than non-reproductive ones. Our SMI.S line separated almost 70% of the reproductive females. Based on our results, we can guarantee that a female whose body condition is above the line will reproduce, although not all those females below the line are non-reproductive, as a few of those under the line will nest. With this information, we have one more biological indicator to take into account when making management and conservation decisions.

## 1. Introduction

The life history of species represents the strategies by which individuals acquire or invest energy during their life cycle [1,2]. The resources available in the environment are limited for animals, so they must optimize their consumption and ensure the allocation of energy to physiological processes and behaviors related to reproduction [3,4], survival, and body growth [2,5,6]. In fact, reproduction has a high metabolic expenditure and demands a high amount of energy [1,7]. As a result of this high metabolic expenditure on reproduction, specialists divide species according to their energy allocation strategy into two categories: capital breeders and income breeders. The first ones accumulate energy that they will then invest in reproduction, while the latter will spend energy on reproduction at the same time as acquiring energy. These categories represent the extremes of the strategy, but individuals can be more or less close to them.

It is generally speculated that ectothermic animals such as reptiles in general, and crocodilians in particular, are highly dependent on capital reproduction [1,8,9]. Therefore, studying the mechanisms and patterns of energy allocation to reproduction can provide valuable information on the life history of these reptile species. It is possible that not all females allocate energy in the same way. It has been reported that there is a strong relationship between the weight and size of the clutch and the females’ sizes [10,11,12], so that the metabolic cost of reproduction would not be the same among adult females. Leiva et al. [8] even mentioned that in unfavorable years, in *Caiman latirostris*, only the larger females are able to reproduce, making it possible to categorize reproductive fitness according to the animal’s size. The species in our study is *Caiman latirostris*, which inhabits warm to temperate climates, in Santa Fe province (Argentina). In this country, it breeds only once a year, in the warm season (December–February), building mound nests and laying an average of 33 eggs (S.E = 5.19 [13]). Incubation lasts approximately 70 days, and during this period, the nesting female protects and maintains the nest [14,15]. The frequency of reproduction of these animals is not reliably known, although a study carried out over two consecutive years indicated that they reproduce every 2 to 4 years (between 30 and 50% of the population are reproductive individuals) [16]. For these animals, the “decision” to breed is likely to depend on the reserves acquired prior to breeding, which should exceed a minimum value.

One way to measure the energy reserve and vigor of an individual is by assessing the body condition of the individual [17,18]. Since in many species good body condition is necessary for reproduction, body condition indices can indicate potential reproductive performance [19]. These indices have the advantage of being easily calculated using morphometric variables such as mass and some measure of body length [20,21,22,23]. This makes them non-invasive, simple and inexpensive to measure. At present, there are several body condition indices (e.g., K-Fulton, SMI, regression), and the choice is related to different criteria, as they are rooted in the field of study, making comparison with other research possible. In crocodilian ecology, the K-Fulton is generally the most widely used [5,24,25,26,27,28,29], although it is gradually migrating to more appropriate indices such as the SMI [8,24,30].

In this context, body condition indices are useful and used in several crocodile studies, where these indices are related to environmental variables [8,28,31,32], to life history patterns–reproduction [3,8], survival, health [24], juvenile migration [3,33], and to ecological interactions–parasite load, social dominance, diet [34], and density [35]. Indices have also been used to evaluate ecological restoration programs and the management of natural populations [26,36].

Therefore, the use of body condition indices can provide valuable information on the life history of these reptile species. In this sense, body condition indices are good indicators of reproductive capacity, as they provide information on the fitness of individuals [3,5,8,37]. Also, the existence of a threshold in the body condition indices (a minimum level of body reserves), which is necessary for reproduction, could show the link between the storage of maternal reserves and the ability to reproduce [38]. Assuming that *Caiman latirostris* can be considered a capital breeder (where reproduction depends on the energy reserves they manage to incorporate prior to reproduction) [1,8,9], there should be a difference in body condition between reproductive and non-reproductive females. If so, we wanted to identify the relationship between maternal restocking and reproductive capacity [39]. Therefore, the aim of the present work was to evaluate whether there is a minimum body condition to trigger reproduction in a female caiman (*Caiman latirostris*) and whether this body condition is related to body size. To do so, we calculated the two body condition indices (K-Fulton and SMI) of adult females (68 < CIII < 99.9 cm snout-vent length SVL; [40]), both in reproductive and non-reproductive states, to determine whether there are differences in body condition between both states.

## 2. Materials and Methods

We obtained the weight and snout-vent length (SVL) of reproductive-stage females of *C. latirostris* (n = 87)—considered in this category according to their size range from 68 to 99.9 cm SVL [40]—from the database of captures carried out by the conservation and sustainable use program Proyecto Yacaré (Santa Fe, Argentina). These captures were performed during a field survey from 2001 to 2018 (with the exception of 2009, because there were no records). All the animal manipulation procedures accorded with the reference guidelines for research with laboratory, farm and wild animals from the National Scientific and Technical Research Council of Argentina [41]. To perform the analyses, we classified the females into two groups according to their reproductive status: (1) non-reproductive (n = 21), referring to females in which no active reproductive structures were observed on the ultrasound scans [16]; and (2) reproductive (n = 66), comprising both gravid females (n = 16), which presented reproductive structures on ultrasound scans showing well-developed vitellogenic follicles, >1.5 cm in diameter and eggs, >4 cm in diameter; (see, for more detail, [16]), as well as females that protected the nest when we approached to capture them (n = 50). For this study, we considered only data from females protecting the nest that had nest clutch size data. For this, we assumed that the female protecting the nest was the nesting female based on genetic studies [14,15]. It is worth mentioning that during data collection, we captured females that lived their entire lives in the wild (n = 67) as well as females released by Proyecto Yacaré (n = 20).

Taking into account that (1) in this study, we evaluated the energy threshold that the female has to achieve in order to trigger reproduction (measured as her body condition); and (2) the females guarding the nest had already made the energetic investment for the production of their eggs, we considered that it was necessary to propose scenarios where the energy already invested is included in the weight of these females. For this reason, the average dry weight of the clutch was included in the weight of females guarding the nest for the calculation of the body condition index. Thus, all the analyses were performed in two scenarios: with the clutch weight and without the clutch weight for the females that were protecting the nest.

For the estimation of the dry weight, we used the following formula and the data derived from Table 1 of Leiva et al. [42]:

$$Clutch\;dry\;weight=CS\times WE\;[Eh+Al\times \left(1-Alm\right)+Y\times \left(1-Ym\right)]\;$$

where *CS* is the clutch size; *WE* is the average weight of each egg (66 g or 0.066 kg); *Eh* is the eggshell ratio (0.192), *Al* is the average egg albumin ratio (0.271); *Alm* is the average albumen moisture ratio (0.949); *Y* is the average yolk ratio (0.539) and *Ym* is the yolk moisture (0.4616). Thus, the added weight represented, on average, 5.93 ± 1.34% of the weight of the females evaluated.

With the SVL (cm) and weight (g) data, we estimated two body condition indices: the scaled mass index (SMI) [21,22] and K-Fulton. It is worth mentioning that the SMI assumes that body condition is independent of animal size, ontogeny and the sex of individuals.

$${SMI}_{i}={M_{i}\times \left[\frac{L0\;}{{SVL}_{i}}\right]}^{b_{SMA}}$$

where $M_{i}$ is the mass of the individual, L0 is the standard size (in this case, we used 100 cm), ${SVL}_{i}$ is the snout-vent length of the individual *i*, and $b_{SMA}$ is the slope of the ordinary least squares regression between the ln ($M_{i}$) and ln (${SVL}_{i}).$ In contrast, the K-Fulton index assumes isometric growth and that body condition does not vary as the animal develops:

$$\;\;K_{i}=10,000\times [\frac{M_{i}}{{{SVL}_{i}}^{3}}]$$

To determine whether there were differences in the body condition index (for both the K-Fulton and SMI) between the non-reproductive and reproductive state groups (both with the estimated clutch weight added–SMI.S and K-Fulton.S—and without the addition), we applied the Wilcoxon test because the data did not meet the homoscedasticity requirement and the number of samples at the factor level was unbalanced.

On the other hand, to estimate the minimum body condition at which females are strictly reproductive, we calculated the function of the SVL-dependent line. To do this, we searched the data for the two non-reproductive females with the highest body condition index, one with the smallest size (Point 1) and the other with the largest size (Point 2). With these two points, we generated the function of the line, calculating the slope and the ordinate to the origin, according to Equations (1) and (2). Each point was worked as an ordered pair, where *y* = body condition index, and *x* = SVL.

(1) $$\mathrm{Slope}:\;a=\frac{y_{2}-y_{1}}{x_{2}-x_{1}}$$

(2) $$\mathrm{Ordered}\;\mathrm{at}\;\mathrm{origin}:\;b=y_{1}-a\;x_{1}$$

Once these values for the equation of the line were obtained, the proportion of individuals R remaining “above” the line was evaluated. Finally, we evaluated whether it was possible to define a threshold body size at which the largest number of reproductive females with body condition below the threshold (TC) could be grouped. Once the TC was selected, we assessed using the Chi-square test whether females larger and smaller than the TC were homogeneously grouped on each side of the threshold, both above and below the body condition line.

## 3. Results

Reproductive females had a higher body condition index than non-reproductive (approximately 15%, Table 1) in both indices, both when body weight alone was taken into consideration (SMI: W = 438; *p* = 0.010; K-Fulton: W = 458.5; *p* = 0.0009) and when estimated clutch weight was added (SMI.S: W = 400; *p* = 0.003; K-Fulton.S: W = 312; *p* < 0.0001).

**Figure 1:**
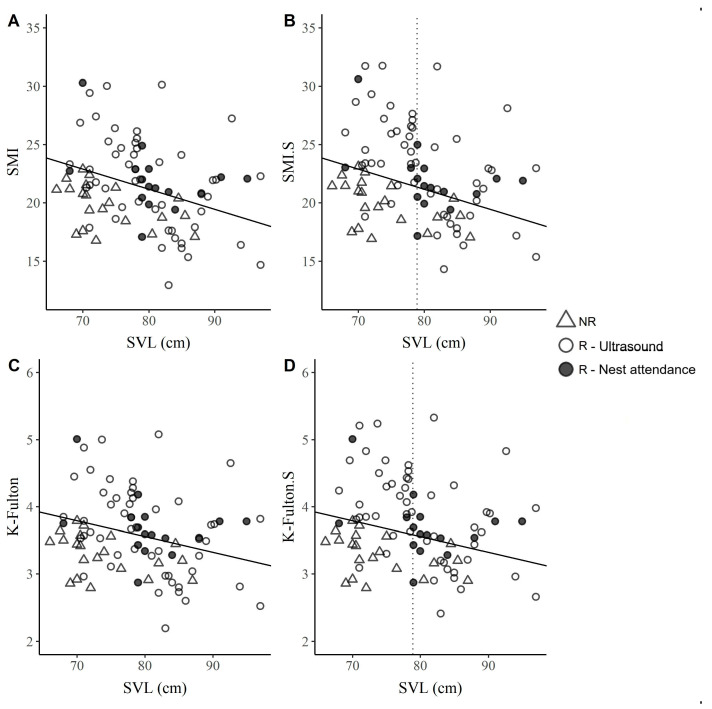
Body condition indices according to body size (snout vent length; SVL) for the 87 females analyzed. The straight lines delimit the body condition of females of a given size that are estimated as strictly reproductive (located above the line) from those that may or may not be reproductive with similar size: (**A**) Scaled mass index (SMI), without the addition of the estimated weight of their eggs to the breeding females attending the nest; (**B**) SMI.S, with the addition of the clutch dry weight to the weight of the clutch found attending the nest; (**C**) K-Fulton, without the addition of the weight of the clutch attending the nest. (**D**) K-Fulton.S, with the addition of the clutch dry weight to the weight of the reproductive females found attending the nest. The color patterns represent the status of the female, filled circle: Reproductive (R) detected via ultrasound; unfilled circle: Reproductive (R) detected attending the nest; triangle: Non-reproductive (NR). The vertical dotted line represents the size (79 cm SVL) at which 80% of the females below the line were found to be reproductive.

The function of the straight line separating the body condition of strictly reproductive females from those that may or may not be reproductive for the data considering only body weight was SMI = −0.17 × SVL + 34.94, and K-Fulton = −0.023 × SVL + 5.44. Above this line were 53% of the reproductive females (40 individuals) for both indices (Figure 1). In the data with the estimated clutch weight added, the line function had 68% (51 individuals) of the reproductive females above the line for both indices (SMI.S = −0.19 × SVL + 36.45, while for the K-Fulton, the function was the same as without adding the weight). There were 20 reproductive females below the line (for both SMI.S and K-Fulton.S); of these, 80% (16 of 20 individuals) had an SVL size >79 cm (TC-X-squared = 6.0984, df = 1, *p* = 0.01). On the other hand, all the non-reproductive females were below the line for both indices.

## 4. Discussion

The body condition indices made it possible to identify differences between reproductive and non-reproductive females, being higher in reproductive females than in non-reproductive females. The higher body condition indices found via both methods in reproductive females, compared to non-reproductive females, may be associated with the storage of energy to be invested later in the progeny. The fact that reproductive females have a higher body condition could indicate that the induction of vitellogenesis requires good physiological and, therefore, body condition to be able to allocate sufficient energy to the production of offspring [39], thus supporting the hypothesis that *Caiman latirostris* could be a largely capital breeder species. With this work, we seek to define the minimum body condition that allows us to differentiate a female that will reproduce from one that will not. We assumed in this study that *C. latirostris* females are capital breeders, i.e., they would invest their own reserves (previously acquired capital) mainly in the production of offspring. To do so, they should reach a minimum level of stored energy for reproduction to occur [38,43], as is the case in *Vipera aspis*, where there is a constant body condition threshold for all body sizes [39]. Although reproduction in crocodilians is size-dependent [12], and considering that the minimum age and size where it occurs in *C. latirostris* are known [40,44,45], we did not observe a fixed or static minimum body condition threshold for the species. The antagonistic strategy to capital breeding is income breeding, whose reproductive costs are met with resources acquired from the environment during the breeding period [38,43]. In fact, both strategies are two antagonistic ends of a continuum, so perhaps *C. latirostris* has a combined strategy between capital and income breeding. It is worth mentioning that this mechanism of combined maternal investment was also previously proposed for wild reproductive females of *C. latirostris*, relating climatic variables (as an indirect indicator of resources in the environment) with body and physiological condition and their reproductive performance [8]. This showed that the energy invested in reproduction comes from a combined strategy, using both stored energy (endogenous origin) and more recently acquired energy.

Although it was not possible to determine a minimum threshold of body condition that relates to the reproductive status of females, we were able to define a linear function that represents the minimum body condition to consider them strictly reproductive, with approximately 30% of the reproductive females remaining below the line determined by the function we found here. These reproductive females with lower body condition could be considered as suboptimal individuals, i.e., with lower reproductive capacity compared to those of the same size. For *C. latirostris*, it has been reported that the higher the body condition of the reproductive female, the higher the hatching success [8]. Based on this, we suppose that females with a body condition lower than that determined by the function found, but that manage to reproduce, i.e., the suboptimal ones, would be doing so to the detriment of the quality of their eggs and, consequently, of their progeny.

If there is some kind of minimum body condition threshold for females, it may not be a fixed value for each size. Madsen and Shine [46] mentioned two hypotheses about the mechanisms by which the reproductive threshold of a population might not be fixed: (1) each individual retains an unchanging threshold, but the composition of the breeding population changes over time, thus varying the population threshold; or (2) individual females show remarkable plasticity and adjust their reproductive threshold to the prevailing conditions of the season. Both hypotheses are reasonable for *C. latirostris*.

The first hypothesis suggests that these females have a fixed threshold and always need a certain body condition to achieve reproduction. For example, it could be that groups of females (perhaps age groups, particular sizes, or genetic subsets of the population) differ in the thresholds and in the conditions necessary to achieve reproduction. We know that only 30% to 50% of adult *C. latirostris* females reproduce per year [47], which indicates that each female reproduces every 2 to 3 years. If the frequency of reproduction is intrinsic to each female, there may be a different combination of females reproducing each year.

In relation to hypothesis 2, there are reports that higher rainfall in March produces an increase in the number of *C. latirostris* nests in the corresponding breeding season [9]. This would indicate that, in those years when conditions are extremely favorable, females would be able to modify their reproductive threshold, supporting the hypothesis of reproductive plasticity. Unfortunately, we do not have recaptures of the same reproductive female in consecutive years, which prevents us from having a better perspective on the reproductive strategies.

In our work, 80% (16 of 20 individuals) of the suboptimal females that managed to reproduce had SVL sizes >79 cm (for the body condition indices with weight added), which could be considered a threshold size (Figure 1). Thus, it is possible that female size is related to breeding frequency, as it is with reproductive characteristics such as egg size or clutch mass [10,12]. However, for reproduction to occur, it is necessary for the physiological condition of the female to allow it [8], so it is possible that not all the size range has the same reproductive capacity, even more so if it is taken into account that the species is physiologically capable of reproducing with an SVL size >69 cm [16,40].

Based on what was reported in our study, assuming that it is correct to add the dry weight of the clutch in females that have already oviposited and taking into account the size of the suboptimal females, it would be possible to divide the females into two categories, depending on whether their size is greater or equal to a 79.1 cm SVL, or less than that value. This division agrees with that postulated by Portelinha et al. [16], who reported that 85% of *C. latirostris* reproductive females were larger than 77 cm, while 75% of non-reproductive ones were smaller. On the other hand, considering that reproduction requires high energy expenditure [7,48] and that this can compete with energy allocation to growth [49], it is possible that females smaller than 79 cm SVL need to acquire energy for further growth, and only in extremely good years are they able to reproduce. In addition, the suboptimal body condition females—larger than a 79.1 cm SVL that managed to reproduce, despite not having optimal reserves, can afford the energy expenditure required to reproduce thanks to being larger.

In order to propose management plans or programs concerning crocodilian species for sustainable use or conservation purposes, it is essential to know how these populations are constituted. By means of different monitoring methods and taking into account the size of the individuals, a population could be described; however, this would not be enough to distinguish the reproductive females from those that are not. In the case of *C. latirostris*, all the potentially reproductive females are in class III > 69 cm SVL [40]. Previous modeling studies of *C. latirostris* proposed a classification of adult females according to clutch size and reproductive probability [50]. In fact, these models suggest that larger females have a greater effect on population dynamics (elasticity values of 0.4) with respect to those of smaller size (0.21). It should be noted that larger females (>79 cm) would generate the greatest contribution to reproduction. Therefore, having information such as the size and body condition of the females would make it possible to have one more biological indicator to take into account when making decisions. This knowledge will make it possible to understand which proportion is generating the greatest contribution to the populations, not only how many potentially reproductive females there are.

This study allowed us to explore the reproduction of *C. latirostris* females and the relationship between size and body condition. However, we found that body condition and female size alone are not a good predictor of female reproductive status. Our study categorized 32% of the females that were reproductive as non-reproductive. Therefore, it may be necessary to generate new studies that include females’ body condition in different years, their size, the availability of prey, and other intrinsic factors related to the animals (number of eggs, clutch quality), which will allow us to understand the reasons for the reported variability. On the other hand, we tried to understand the difference in body condition using two different body condition index (K-Fulton and SMI). Currently, there is no consensus on the best method of calculating the body condition index, or on the criteria to be used, and few authors provide a detailed justification for their choice [51]. In general, ecologists choose to use the method that is the most widely established in their field of study, thus allowing for direct comparisons between populations, species, or reproductive stages [51]. For example, the K-Fulton index is used historically in crocodilians [25,36,52], although at this time, some work is migrating to other indices, such as the SMI [8,30,32]. This change is because conventional body condition indices may be inherently biased with respect to animal size and tend to accentuate the condition values of larger animals because they do not meet animal isometry assumptions [21,22,53]. However, here, we analyzed data from the same category or class: adult females. Therefore, the effect of the animal’s size/area ratio and allometric growth would not be influencing the proportional change in the indices. Therefore, although the numerical results between the two groups are different, the changes as the SVL of the animals’ increases are barely perceptible. This renders it not essential to report both indices, although recording them may generate an interesting accumulation of information to be compared in the future and thus change a simpler index for a more adequate one.

## 5. Conclusions

We studied the stored energy, measured as the body condition, of reproductive and non-reproductive females. We found that although reproductive adult females have higher body condition than non-reproductive females, stored energy appears to be a necessary but not exclusive condition for initiating breeding, because we did not find a threshold for reproductive condition. However, females larger than 79 cm SVL with low body condition would reproduce. This would indicate that smaller caimans would require a greater concentration and investment of energy to reproduce. Therefore, categorizing adult females by body size and condition provides an important biological indicator for management and conservation decisions.

## Figures and Tables

**Table 1:** SMI and K-Fulton body condition indexes for reproductive and non-reproductive females. Scaled mass index (SMI) and K-Fulton: Indices based on the body weight of the individual without the addition of the dry weight of her clutch; SMI.S and K-Fulton.S: SMI and K-Fulton indices calculated by adding the average dry weight of her clutch to the body weight of the female attending the nest.

Status	Index	Mean	SD	Median	Range
Reproductive (n = 75)	SMI.S	22.88	4.04	22.95	14.31–31.73
	SMI	21.79	3.8	21.9	12.95–30.27
	K-Fulton.S	3.83	0.65	3.83	2.41–5.33
	K-Fulton	3.66	0.62	3.68	2.19–5.08
Non-reproductive (n = 21)	SMI	19.77	1.87	19.99	16.77–22.88
	K-Fulton	3.29	0.3	3.33	2.79–3.79

## Data Availability

The data presented in this study are available on request from the corresponding author. The data are not publicly available due to because they are linked to the work of other members of the group, and must be presented as part of doctoral theses according to university regulations.

## References

[1] Bonnet X. (1998). Capital versus Income Breeding: An Ectothermic Perspective. Oikos.

[2] Healy K., Ezard T.H.G., Jones O.R., Salguero-Gómez R., Buckley Y.M. (2019). Animal Life History Is Shaped by the Pace of Life and the Distribution of Age-Specific Mortality and Reproduction. Nat. Ecol. Evol..

[3] Barão-Nóbrega J.A.L., Marioni B., Botero-Arias R., Nogueira A.J.A., Lima E.S., Magnusson W.E., Da Silveira R., Marcon J.L. (2018). The Metabolic Cost of Nesting: Body Condition and Blood Parameters of *Caiman crocodilus* and *Melanosuchus niger* in Central Amazonia. J. Comp. Physiol. B Biochem. Syst. Environ. Physiol..

[4] Shine R. (1980). “Costs” of Reproduction in Reptiles. Oecologia.

[5] Mazzotti F.J., Cherkiss M.S., Brandt L.A., Fujisaki I., Hart K., Jeffery B., McMurry S.T., Platt S.G., Rainwater T.R., Vinci J. (2012). Body Condition of Morelet’s Crocodiles (*Crocodylus moreletii*) from Northern Belize. J. Herpetol..

[6] Zera A.J., Harshman L.G. (2001). Physiology of Life History Trade-Offs in Animals. Annu. Rev. Ecol. Evol. Syst..

[7] French S.S., Virgin E.E., Ki K.C., Maryon D.F., Goode A.B.C., Pasachnik S.A. (2021). Reproductive Stage and Clutch Size Incur Energetic and Oxidative Costs in an Endangered Iguana, *Ctenosaura oedirhina*. J. Herpetol..

[8] Leiva P.M.L., Labaque M.C., Piña C.I., Simoncini M.S. (2023). Influence of Climatic Variables on Corporal Attributes of Adult Female Caiman and Their Relationship with Reproductive Success. S. Am. J. Herpetol..

[9] Simoncini M.S., Piña C.I., Cruz F.B., Larriera A. (2011). Climatic Effects on the Reproductive Biology of *Caiman latirostris* (Crocodylia: Alligatoridae). Amphib. Reptil..

[10] Larriera A., Piña C.I., Siroski P., Verdade L.M. (2004). Allometry of Reproduction in Wild Broad-Snouted Caimans (*Caiman latirostris*). J. Herpetol..

[11] Thorbjarnarson J.B. (1996). Reproductive Characteristics of the Order Crocodylia. Herpetologica.

[12] Verdade L.M. (2001). Allometry of Reproduction in Broad-Snouted Caiman (*Caiman latirostris*). Braz. J. Biol..

[13] Simoncini M.S., Piña C.I., Siroski P.A. (2009). Clutch Size of *Caiman latirostris* (Crocodylia: Alligatoridae) Varies on a Latitudinal Gradient. North-West. J. Zool..

[14] Amavet P., Rosso E., Markariani R., Piña C.I. (2008). Microsatellite DNA Markers Applied to Detection of Multiple Paternity in *Caiman latirostris* in Santa Fe, Argentina. J. Exp. Zool. Part A Ecol. Genet. Physiol..

[15] Amavet P.S., Vilardi J.C., Rueda E.C., Larriera A., Saidman B.O. (2012). Mating System and Population Analysis of the Broad-Snouted Caiman (*Caiman latirostris*) Using Microsatellite Markers. Amphib. Reptil..

[16] Portelinha T.C.G., Jahn G.A., Hapon M.B., Verdade L.M., Piña C.I. (2015). Hormone Levels and Ultrasound Evaluation of *Caiman latirostris* (Crocodylia, Alligatoridae) Ovulation. S. Am. J. Herpetol..

[17] Arcos-García J.L., Ordaz J.N., Grajales J.G., Zozaya R.D.P.R., Romero H.S., Pozos R.L. (2020). Body Condition Index in Breeding Black Iguana Females (*Ctenosaura pectinata*) in Captivity. Rev. Fac. Cienc. Agrar..

[18] Milenkaya O., Catlin D.H., Legge S., Walters J.R. (2015). Body Condition Indices Predict Reproductive Success but Not Survival in a Sedentary, Tropical Bird. PLoS ONE.

[19] Waye H.L., Mason R.T. (2008). A Combination of Body Condition Measurements Is More Informative than Conventional Condition Indices: Temporal Variation in Body Condition and Corticosterone in Brown Tree Snakes (*Boiga irregularis*). Gen. Comp. Endocrinol..

[20] Labocha M.K., Schutz H., Hayes J.P. (2014). Which Body Condition Index Is Best?. Oikos.

[21] Peig J., Green A.J. (2010). The Paradigm of Body Condition: A Critical Reappraisal of Current Methods Based on Mass and Length. Funct. Ecol..

[22] Peig J., Green A.J. (2009). New Perspectives for Estimating Body Condition from Mass/Length Data: The Scaled Mass Index as an Alternative Method. Oikos.

[23] Wilder S.M., Raubenheimer D., Simpson S.J. (2016). Moving beyond Body Condition Indices as an Estimate of Fitness in Ecological and Evolutionary Studies. Funct. Ecol..

[24] Balaguera-Reina S.A., Brandt L.A., Hernandez N.D., Mason M., Smith C.D., Mazzotti F.J. (2023). Body Condition as a Descriptor of American Alligator (*Alligator mississippiensis*) Health Status in the Greater Everglades, Florida, United States. PLoS ONE.

[25] Brandt L.A., Nestler J.H., Brunell A.M., Beauchamp J.S., Mazzotti F.J., Nestler J.H., Brunell A.M., Beauchamp J.S., Mazzotti F.J. (2016). Variation in Body Condition of *Alligator mississippiensis* in Florida. Bull. Fla. Mus. Nat. Hist..

[26] Briggs-Gonzalez V.S., Basille M., Cherkiss M.S., Mazzotti F.J. (2021). American Crocodiles (*Crocodylus acutus*) as Restoration Bioindicators in the Florida Everglades. PLoS ONE.

[27] Buenfil-Rojas A.M., Alvarez-Legorreta T., Cedeño-Vázquez J.R. (2018). Mercury and Metallothioneins in Blood Fractions and Tissues of Captive Morelet’s Crocodiles in Quintana Roo, Mexico. Chemosphere.

[28] Fujisaki I., Rice K.G., Pearlstine L.G., Mazzotti F.J. (2009). Relationship between Body Condition of American Alligators and Water Depth in the Everglades, Florida. Hydrobiologia.

[29] Webb E.C., Veldsman D.M., Myburgh J.G., Swan G.E. (2021). Effects of Stocking Density on Growth and Skin Quality of Grower Nile Crocodiles (*Crocodylus niloticus*). S. Afr. J. Anim. Sci..

[30] Ojeda-Adame R.A., Hernández-Hurtado H., Ramírez-Martinez M.M., Iñiguez-Davalos L.I. (2020). A Body Condition Score for Crocodilians. S. Am. J. Herpetol..

[31] Brandt L.A., Beauchamp J.S., Jeffery B.M., Cherkiss M.S., Mazzotti F.J. (2016). Fluctuating Water Depths Affect American Alligator (*Alligator mississippiensis*) Body Condition in the Everglades, Florida, USA. Ecol. Indic..

[32] McCaffrey K.R., Balaguera-Reina S.A., Falk B.G., Gati E.V., Cole J.M., Mazzotti F.J. (2023). How to Estimate Body Condition in Large Lizards? Argentine Black and White Tegu (*Salvator merianae*, Duméril and Bibron, 1839) as a Case Study. PLoS ONE.

[33] Litzgus J.D., Bolton F., Schulte-Hostedde A.I. (2008). Reproductive Output Depends on Body Condition in Spotted Turtles (*Clemmys guttata*). Copeia.

[34] Delany M.F., Linda S.B., Moore C.B. (1999). Diet and Condition of American Alligators in 4 Florida Lakes. Proceedings of the Annual Conference of the Southeast Association of Fish and Wildlife Agencies.

[35] Padilla S.E., Weber M., Jacobson E.R. (2011). Hematologic and Plasma Biochemical Reference Intervals for Morelet’s Crocodiles (*Crocodylus moreletii*) in the Northern Wetlands of Campeche, Mexico. J. Wildl. Dis..

[36] Mazzotti F.J., Best G.R., Brandt L.A., Cherkiss M.S., Jeffery B.M., Rice K.G. (2009). Alligators and Crocodiles as Indicators for Restoration of Everglades Ecosystems. Ecol. Indic..

[37] Speakman J.R. (2001). Body Composition Analysis of Animals: A Handbook of Non-Destructive Methods.

[38] Jönsson K.I. (1997). Capital and Income Breeding as Alternative Tactics of Resource Use in Reproduction. Oikos.

[39] Naulleau G., Bonnet X. (1996). Body Condition Threshold for Breeding in a Viviparous Snake. Oecologia.

[40] Leiva P.M.L., Simoncini M.S., Portelinha T.C.G., Larriera A., Piña C.I. (2019). Size of Nesting Female Broad-Snouted Caimans (*Caiman latirostris* Daudin 1802). Braz. J. Biol..

[41] National Scientific and Technical Research Council (2005). Reference Ethical Framework for Biomedics Research: Ethical Principles for Research with Laboratory, Farm and Wild Animals.

[42] Leiva P.M.L., Labaque M.C., Fernandez M.E., Piña C.I., Simoncini M.S. (2018). Physical and Chemical Characteristics of Fertile and Infertile Eggs of Wild *Caiman latirostris*. Aquaculture.

[43] Stephens P.A., Boyd L., McNamara J.M., Houston A. (2009). Capital Breeding and Income Breeding: Their Meaning, Measurement, and Worth. Ecology.

[44] Larriera A., Siroski P., Piña C.I., Imhof A. (2006). Sexual Maturity of Farm-Released *Caiman latirostris* (Crocodylia: Alligatoridae) in the Wild. Herpetol. Rev..

[45] Verdade L.M., Sarkis-Gonçalves F., Miranda-Vilela M.P., Bassetti L.A.B. (2003). New Record of Age at Sexual Maturity in Captivity for *Caiman latirostris* (Broad-Snouted Caiman). Herpetol. Rev..

[46] Madsen T., Shine R. (1999). The Adjustment of Reproductive Threshold to Prey Abundance in a Capital Breeder. J. Anim. Ecol..

[47] Portelinha T.C.G. (2016). Área de Vida, Uso de Hábitat y Ciclo Reproductivo de *Caiman latirostris* (Crocodylia: Alligatoridae) En La Provincia de Santa Fe, Argentina. Ph.D. Thesis.

[48] Webb A.C., Iverson J.B., Knapp C.R., DeNardo D.F., French S.S. (2019). Energetic Investment Associated with Vitellogenesis Induces an Oxidative Cost of Reproduction. J. Anim. Ecol..

[49] Kubička L., Kratochvíl L. (2009). First Grow, Then Breed and Finally Get Fat: Hierarchical Allocation to Life-History Traits in a Lizard with Invariant Clutch Size. Funct. Ecol..

[50] Viotto E.V., Navarro J.L., Simoncini M.S., Piña C.I. (2023). Stage-Based Model of Population Dynamics and Harvest of Broad-Snouted Caiman (*Caiman latirostris*) under Different Management Scenarios. Ethnobiol. Conserv..

[51] Stevenson R.D., Woods W.A. (2006). Condition Indices for Conservation: New Uses for Evolving Tools. Integr. Comp. Biol..

[52] Tellez M., Arevalo B., Paquet-Durand I., Heflick S. (2017). Population Status of Morelet’s Crocodile (*Crocodylus moreletii*) in Chiquibul Forest, Belize. Mesoam. Herpetol..

[53] Falk B.G., Snow R.W., Reed R.N. (2017). A Validation of 11 Body-Condition Indices in a Giant Snake Species That Exhibits Positive Allometry. PLoS ONE.

